# Maresin: Macrophage Mediator for Resolving Inflammation and Bridging Tissue Regeneration—A System-Based Preclinical Systematic Review

**DOI:** 10.3390/ijms241311012

**Published:** 2023-07-02

**Authors:** Wen-Chun Liu, Yu-Hsin Yang, Yu-Chin Wang, Wei-Ming Chang, Chin-Wei Wang

**Affiliations:** 1 School of Dentistry, College of Oral Medicine, Taipei Medical University, No. 250, Wuxing St., Taipei 110310, Taiwan; b202108042@tmu.edu.tw (W.-C.L.); b202108010@tmu.edu.tw (Y.-H.Y.); b202107096@tmu.edu.tw (Y.-C.W.); 2 School of Oral Hygiene, College of Oral Medicine, Taipei Medical University, Taipei 110301, Taiwan; weiminchang@tmu.edu.tw; 3 Division of Periodontics, Department of Dentistry, Taipei Medical University Hospital, Taipei 110301, Taiwan

**Keywords:** maresin, resolving inflammation, tissue regeneration, preclinical studies, human organ systems

## Abstract

Maresins are lipid mediators derived from omega-3 fatty acids with anti-inflammatory and pro-resolving properties, capable of promoting tissue regeneration and potentially serving as a therapeutic agent for chronic inflammatory diseases. The aim of this review was to systematically investigate preclinical and clinical studies on maresin to inform translational research. Two independent reviewers performed comprehensive searches with the term “Maresin (NOT) Review” on PubMed. A total of 137 studies were included and categorized into 11 human organ systems. Data pertinent to clinical translation were specifically extracted, including delivery methods, optimal dose response, and specific functional efficacy. Maresins generally exhibit efficacy in treating inflammatory diseases, attenuating inflammation, protecting organs, and promoting tissue regeneration, mostly in rodent preclinical models. The nervous system has the highest number of original studies (*n* = 25), followed by the cardiovascular system, digestive system, and respiratory system, each having the second highest number of studies (*n* = 18) in the field. Most studies considered systemic delivery with an optimal dose response for mouse animal models ranging from 4 to 25 μg/kg or 2 to 200 ng via intraperitoneal or intravenous injection respectively, whereas human in vitro studies ranged between 1 and 10 nM. Although there has been no human interventional clinical trial yet, the levels of MaR1 in human tissue fluid can potentially serve as biomarkers, including salivary samples for predicting the occurrence of cardiovascular diseases and periodontal diseases; plasma and synovial fluid levels of MaR1 can be associated with treatment response and defining pathotypes of rheumatoid arthritis. Maresins exhibit great potency in resolving disease inflammation and bridging tissue regeneration in preclinical models, and future translational development is warranted.

## 1. Introduction

Maresins are lipid mediators and members of the omega-3 fatty acid derivatives having both anti-inflammatory and pro-resolving abilities, including maresin 1 (7,14-dihydroxydocosa-4Z,8,10,12,16Z,19Z-hexaenoic acid, MaR1), maresin 2 (13R,14S-dihydroxy-docosahexaenoic acid, MaR2) [1,2,3,4], and maresin conjugate in tissue regeneration (MCTR). Maresin 1 was first identified in self-limiting peritonitis exudate in the murine model, and later, total organic synthesis was achieved. It was also found in several human tissue fluids and that human macrophage can produce MaR1. This process involves the conversion of DHA to 14S-HpDHA, with 12-lipoxygenase (12-LOX) being the main enzyme involved. Subsequently, the epoxidation of 14S-HpDHA leads to the formation of 13S,14S-epoxy-maresin. Finally, hydrolysis of 13S,14S-epoxy-maresin takes place to complete the pathway [5]. Interestingly, the level of 12-LOX was found to be highest in the M2 macrophage and dendritic cells [4]. MaR1 can reduce polymorphonuclear leukocyte (PMN) infiltration in zymosan-induced acute peritonitis, promote macrophage phagocytosis of apoptotic PMNs and efferocytosis, stimulate tissue regeneration in planaria (MaR1 optimal: 100 nM), and inhibit pain [1,2,3]. It regulates human phagocytes through the leucine-rich repeat-containing G protein-coupled receptor 6 (LGR6) pathway to activate the pre-resolving phase of inflammation [6]. Additionally, MaR2 [4] and maresin conjugates in tissue regeneration (MCTR) [7,8] were also found to be bioactive. Despite there being a fewer number of studies on MaR2 and MCTR, they should have their own unique role and potential that is yet to be explored.

When the body responds to pathogens, trauma, or tissue damage, inflammatory responses are driven by pro-inflammatory mediators and this acute inflammatory response is protective. However, if this progress is not controlled, chronic inflammatory diseases will evolve. In an ideal situation, the stimuli is resolved, and the tissue will gradually return to homeostasis. The resolution of inflammation was once believed to be passive, but now it is found to be an active process, involving lipid mediator class-switching from pro-inflammatory mediators to pro-resolving mediators such as maresin. Therefore, maresins have a vast therapeutic potential to alleviate chronic inflammatory disease. Additionally, since maresin was produced by a macrophage, the main player for tissue healing and regeneration, MaR1 has been shown to promote tissue regeneration in different organs and systems. Although there are many drugs available to suppress inflammation and pain, such as corticosteroids and NSAIDs, they are accompanied by side effects that can be harmful to the body in the long term [9,10]. Natural pro-resolving signals and molecules such as maresins should hold huge potential with limited adverse effects to control chronic inflammatory diseases.

**Figure 1:**
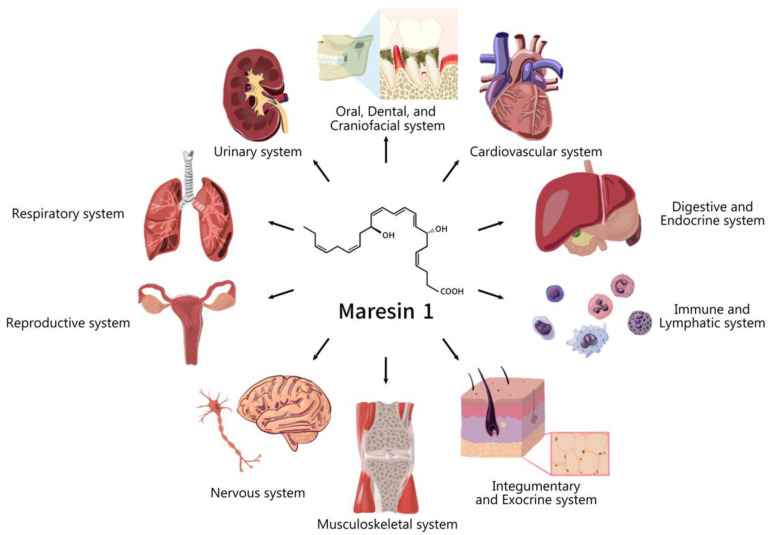
Human organ systems affected by maresin 1.

To date, an increasing number of preclinical studies have been conducted on maresins; however, there is a lack of systematic synthesis of this translational information. Systematic reviews (SR) are usually conducted for clinical human studies, yet appraising preclinical human and animal studies have their merits and significance in the field of translational medicine. They can avoid redundancies of experiments and trials, give inferences for future experimental designs, and justify further investigation in clinical trials. Additionally, compared to human clinical trials, animal studies can offer a chance to test critical hypotheses that cannot be tested in humans. Thus, SR of preclinical human and animal studies may give us essential information to advance the field. Therefore, it is crucial to conduct quality systematic reviews on preclinical studies. In this article, we aim to conduct a systematic review of maresins for treating different diseases and conditions in different systems (Figure 1).

## 2. Materials and Methods (Including Flow Diagram)

### 2.1. Search Strategy

This systematic review was conducted following the Reporting Items for Systematic Reviews and Meta-Analyses (PRISMA) guidelines and referring to the Collaborative Approach to Meta-Analysis and Review of Animal Data from Experimental Studies (CAMARADES) [11]. Articles included were results of the search term “Maresin (NOT) Review” before August 2022 in the PubMed database. Our research questions are: “What clinical and preclinical studies have been reported about maresins, and what are the primary or functional outcomes?”

- P (Population): Human or animal with preclinical models.
- I (Intervention): Application of maresins, either locally or systematically.
- C (Comparison): Healthy or vehicle control without intervention.
- (Outcomes): Preclinical or functional outcomes.

### 2.2. Inclusion and Exclusion Criteria

**Figure 2:**
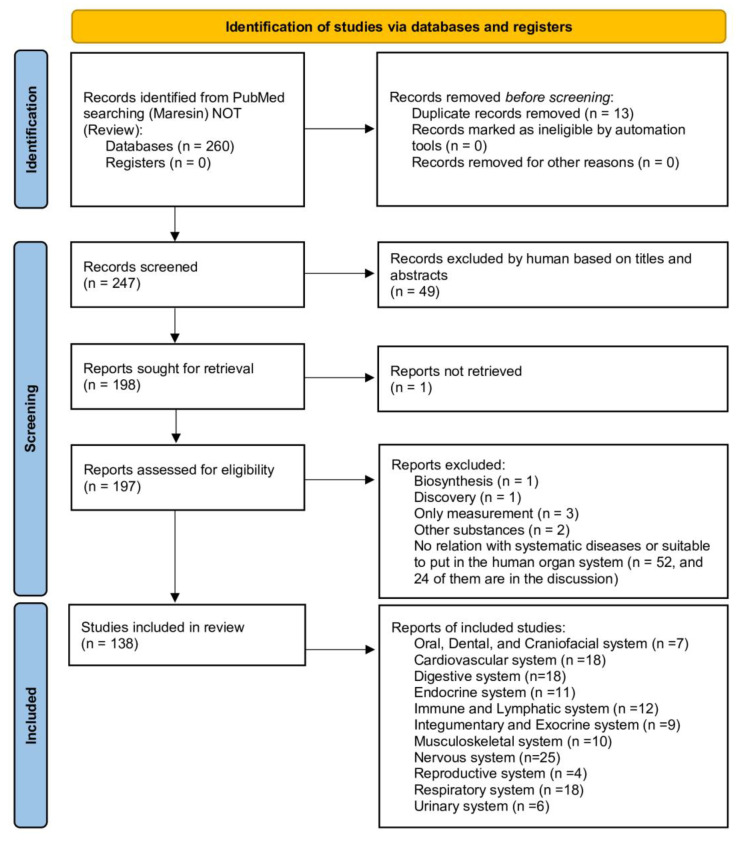
PRISMA flowchart illustrating the article selection process.

Two reviewers, Liu and Yang, screened the data independently and manually. Only original studies published in English that could address the PICO question were considered. Comments, editorials, hypotheses, review articles, and non-preclinical outcome-related research (biosynthesis, chemical structure, chromatography, discovery, drug development, and unrelated to maresin) were excluded based on titles and abstracts. Included articles were read carefully, and some were removed (reasons are in the flow chart). We classified those studies into 11 different categories, depending on the human body’s organ systems. The organ systems are (1) oral, dental, and craniofacial system, (2) cardiovascular system, (3) digestive system, (4) endocrine system, (5) immune and lymphatic system, (6) integumentary and exocrine system, (7) musculoskeletal system, (8) nervous system, (9) reproductive system, (10) respiratory system, (11) urinary system. Where there were different opinions, e.g., articles were classified in different systems, there was uncertainty about which organ systems to categorize, or the articles spanned multiple systems, were determined by discussions with CW. Wang, and all authors agreed with the final decisions, including data and classifications. Detailed processes are presented in Figure 2.

### 2.3. Data Extraction

In this systematic review, we focus on the field of translational medicine and the connections between human organ systems. Therefore, only preclinical studies with systematic diseases were considered. Data from the included studies were extracted from the original main texts or the figures. If there were no raw data, we changed the results into relative proportions. We collated the disease models, the way maresins were administered, optimal concentration, and the effects they brought. Detailed information is provided in Table 1.

## 3. Results and Discussion

### 3.1. Oral, Dental, and Craniofacial System

The oral, dental, and craniofacial system occupies more than half of the skull and coordinates mastication, ingestions, and pronunciation for communication. This system also represents an important individual identity as one smiles. Inflammation in the oral, dental, and craniofacial system, such as, periodontitis, not only influences one’s food intake, pronunciation, and confidence, but it can also influence the risk and aggravation by other systemic chronic inflammatory diseases, e.g., diabetes, arthritis, and cardiovascular diseases [81,82,83,84]. As a result, MaR1, as part of the pro-resolving lipid mediator family, has shown the potential ability to control inflammation and exert its therapeutic benefits.

#### 3.1.1. Periodontal Diseases

Periodontal disease is a chronic inflammatory disease, and patients with aggressive periodontitis (AgP, currently termed Grade C periodontitis) usually present with rapidly progressive alveolar bone loss around dentition. The level of 14-HDHA (hydrolysis product of precursor of MaR1) was found to be higher in the serum of patients with AgP, suggesting that the biosynthetic pathway or MaR1 and metabolites may be dysregulated in patients with AgP [85]. In a more homogeneous cohort of patients with localized aggressive periodontitis (LAP, currently termed Stage IV Grade C molar-incisal pattern periodontitis), MaR1 and the level of 12-lipoxygenase (12-LOX) were found to be lower from macrophages (only 36% and 30% the level of healthy control). Phagocytes of patients with LAP have lower ability in phagocytosis and killing pathogens. However, additional MaR1 administration enhanced phagocytosis (peaks at 1 nM), restored bactericidal capacity, and increased intracellular ROS generation [12]. Exogenous MaR1 also increases healthy human periodontal ligament cells’ (hPDLCs’) survival rate and autophagy. MaR1 also decreases hPDLCs’ apoptosis and inflammatory factors production such as IL-6, IL-8, TNF-α, and IL-1β, under *P. gingivalis* (peaks at 10 nM) stimulation through the activation of the glycogen synthase kinase-3β (GSK-3) and β-catenin pathway [13].

#### 3.1.2. Human Periodontal Ligament Stem Cells (hPDLSCs)

MaR1 can reverse the activation of human periodontal ligament stem cells (hPDLSCs) by TNF-α and IL-1β, which includes the expression of pluripotency, migration, viability, and differentiation into a PDL-like cell phenotype [14]. The study also showed that pig PDLSCs (pPDLSCs) biosynthesize maresin conjugate in tissue regeneration 3 (MCTR3). Under inflammatory conditions, pretreatment of MCTR3 inhibits pPDLSCs from producing prostaglandin E2 (PGE2) in a dose-dependent manner (optimal: 10 nM~100 nM) and reduces the production of IL-6 and IL-8 at the early stage [86].

#### 3.1.3. Tooth Extraction and Temporomandibular Joint (TMJ) Pain

For oral wound healing, such as tooth extraction, topical MaR1 (0.05 μg/μL, delivered through gelatin scaffolds) has the highest potency to promote wound closure and re-epithelialization. MaR1 increases extraction socket bone fill, preserves the alveolar bone ridge, regulates M2-like macrophage surface marker CD206, and reduces post-operative pain [15]. MaR1 (0.35 nM, local injection) also inhibits capsaicin-induced pain in the temporomandibular joint (TMJ) by blocking transient receptor potential vanilloid 1 (TRPV1) current and inhibiting inflammation-generated neuroplasticity [87].

Collectively, maresins have various therapeutic applications in the oral, dental, and craniofacial systems, regulating phagocytes and PDL stem cells. Maresins also have therapeutic potential in treating periodontitis and temporomandibular disorder, as well as promoting the healing and regeneration of oral tissues. Table 1 is the related compilation of preclinical models of the oral, dental, and craniofacial system.

### 3.2. Cardiovascular System

The cardiovascular system is responsible for a large percentage of the total body circulation and is an important pathway linking the other systems. Common diseases of this system include atherosclerosis, myocardial infarction, hypertension, and sepsis. Cardiovascular diseases (CVDs) are largely caused by fatty substances built up in the arteries, and they can increase the risks of blood clots and the above-mentioned major medical adverse events [88,89,90]. Following the published investigations, MaR1 demonstrates potential therapeutic ability in the cardiovascular system.

#### 3.2.1. Cardiomyopathy

In terms of sepsis-induced cardiomyopathy (SIC), maresin conjugate in tissue regeneration 1 (MCTR1, 0.15 nmol/mouse, *i.v.*) was found to reduce neutrophil infiltration and enhance cardiac function via the IL-17 signaling pathway, and also improve mitochondrial biogenesis and function [16,17]. Pretreatment of MaR1 (100 ng, *i.p.*) followed by booster injections ((100 ng per mouse) every 2 days, *i.p.*) to SIC mice showed that MaR1 could protect the heart from injury and dysfunction [18]. In the process of tissue repair, MaR1 induced cardiomyocyte hypertrophy through the IGF-1 paracrine pathway, and the production of IGF-1 was mediated by the RORα/IGF-1/PI3K/Akt pathway. [91].

#### 3.2.2. Sepsis and Pulmonary Arterial Hypertension (PAH)

MaR1 (1.1 ng, *i.v.*) protected mice from lung injury (cecal ligation and puncture, CLP) by inhibiting the activation nuclear factor kappa B (NF-κB) pathway. An amount of 10 nM of MaR1 attenuated mitochondrial dysfunction both in CLP mice and peripheral venous blood from sepsis patients dependent on the lipoxin receptor (ALX) and cAMP [19,20]. Moreover, pulmonary arterial hypertension (PAH) mice have lower endogenous MaR1, and the additional administration of MaR1 (1 μg/mouse, *i.p.*) followed by booster injections (100 ng per mouse) every 2 days lessened right ventricular systolic pressure (RVSP), attenuated right ventricular dysfunction (RVD), reversed abnormal changes in pulmonary vascular remodeling, and inhibited abnormal pulmonary artery smooth muscle cells’ (PASMCs’) proliferation via LGR6 [21].

#### 3.2.3. Atheroprogression, Atherosclerosis, Coronary Artery Diseases, and Vascular Inflammation

MaR1 (100 ng each, *i.p.*) can reduce the risks of developing atheroma, including diminishing lipopolysaccharide (LPS)-induced atherosclerotic reactions and preventing atheroprogression of hypercholesterolemic ApoE^−/−^ mice [22,92]. MaR1 (4 or 40 ng/g bodyweight, *i.p.*) also reduces abdominal aortic aneurysm (AAA) growth of smooth muscle cells in the topical elastase AAA mice model [23]. Women with non-obstructive coronary artery disease (CAD) had lower plasma levels of MaR1. However, after oral administration of EPA + DHA (3.36 g daily), MaR1 was significantly increased in plasma [93,94]. Moreover, MaR1 (10 ng/g, *i.p.*, every 2 days for 28 days) attenuates ventricular remodeling (remaining 52% of myocardial fibrosis area) and reduces arrhythmias in mice after myocardial infarction (MI) by activating the NRF2/HO-1 signaling pathway and inhibiting the TLR4/NF-kB signaling pathway [24]. In vascular inflammation, MaR1 had anti-inflammatory effects on vascular endothelial (EC) and smooth muscle cells (VSMC). In a mouse model of arterial neointima formation (carotid ligation), neointimal hyperplasia was reduced (67%) in MaR1-treated mice (*i.p.*, 100 ng) [95,96]. Moreover, leukotriene-D4-stimulated vascular responses were counter-regulated by MCTR through cysteinyl leukotriene receptor-1 (CysLT1), and MCTR (0.1–10 nM) stimulated human macrophages’ phagocytosis, suggesting that the imbalance of pro-inflammatory and pro-resolving mediators is associated with diseases [8].

#### 3.2.4. Blood Coagulation

The coagulation of human whole blood (WB) generated temporal clusters of endogenously produced prothrombotic and pro-inflammatory lipid mediators (LMs, eicosanoids) and specialized pro-resolving mediators (SPMs, including MaR1). Treatment of SPM clusters improved phagocytosis of leukocytes’ abilities of killing *Escherichia coli* (*E. coli*.) [97]. Interestingly, MaR1 managed the aggregation (approximately 70% at 10 μM, the same as 7-epi-MaR1) and spreading of platelets, as well as suppressed the release of pro-inflammatory and prothrombotic mediators. Notably, 7S,14S-diHDHA demonstrated a strong anti-aggregation ability; it fully inhibited platelet aggregation at 10 μM while reducing aggregation by 65% at 3 μM [98,99].

Generally, MaR1 and its related metabolites have cardiovascular protection functions and/or inhibit the progression of CVDs, and some of them can also prevent the coagulation of blood. Table 1 is the related compilation of preclinical models of the cardiovascular system.

### 3.3. Digestive System

The digestive system is comprised of the gastrointestinal (GI) tract, liver, pancreas, and gallbladder. The digestive system breaks down and absorbs nutrients from food and liquids with enzymes. Inflammatory digestive system diseases such as inflammatory bowel disease (Crohn’s disease and ulcerative colitis), pancreatitis, and liver injury can cause irreversible tissue damage and seriously affect body function, and are potentially life-threatening.

#### 3.3.1. Colitis

Colitis is usually relevant to inflammatory bowel disease (IBD). Maresin 1 (50 μg/kg; oral gavage) can increase the relative abundance of *P. xylanivorans* (a probiotic with healthy effects on gut inflammation) while decreasing the expression of IL-1β and TNF-α in diet-induced obese (DIO) mice [25]. In another murine model experiment, evidence showed that MaR1 improved the disease activity index and reduced body weight and colonic tissue damage, protecting mice against colitis. MaR1 (0.1, 0.3, and 1 μg/animal; *e.v.*) decreased ICAM-1 mRNA expression and the levels of inflammatory cytokines, inhibiting TLR4 and NF-κB pathway and activating Nrf2 signaling. Furthermore, MaR1 enhanced the macrophage M2 phenotype and decreased myeloperoxidase (MPO) activity and reactive oxygen species (ROS) by improving the expression of tight junction proteins and reducing the infiltration of neutrophils and macrophages [26,100].

Approximately 50% of patients with IBD suffer from anemia, and most of these with iron deficiency. MaR1 (0.1, 0.3, 1 ng; *i.p.*) treatment ameliorates iron-deficient anemia by reducing colonic inflammation and inhibiting hepcidin expression through the IL-6/STAT3 pathway dose dependently [101].

#### 3.3.2. Hepatitis and Liver Fibrosis

Hepatitis is one of the most common liver diseases, and liver fibrosis is a result of most types of chronic liver diseases, such as cirrhosis, hepatocellular carcinoma, and liver failure. MaR1 inhibited thiobarbituric acid reactive substances and increased the activities of antioxidative mediators (ROS levels decreased). MaR1 also inhibited NF-κB pathway activation and mitogen-activated protein kinases (MAPKs), showing antioxidative and anti-inflammatory effects, attenuating hepatic injury, oxidative stress, and lipid peroxidation. MaR1 improved liver function by reducing hepatocyte apoptosis and increasing macrophage apoptosis [27,102].

MaR1 can alleviate diethylnitrosamine (DEN)-induced hepatocarcinogenesis. Comparing DEN, MaR1, and MaR1 + DEN groups, respectively, the levels of aspartate transaminase (AST) concentration were 199.7 ± 54.9, 85.5 ± 29.5, and 107.0 ± 28.3; the alanine transaminase (ALT) concentrations were 89.6 ± 40.6, 55.5 ± 33.6, and 50. 4 ± 23.5 (UI/L); and the hepatic indexes were 5.5 ± 0.21, 4.5 ± 0.3, and 4.8 ± 0.15. With DEN stimulation, the pro-inflammatory cytokines TNF-α and IL-1β were increased 5.5-fold in the control group and 4.8- and 2.4-fold in relation to the MaR1 + DEN group, respectively. Meanwhile, the anti-inflammatory IL-10 of the MaR1 + DEN group was 3.5- and 3.4-fold compared to the control and MaR1 group, respectively, and 5.5-fold to the DEN group. The hepatic architecture was improved, and inflammation and necrotic areas were reduced [28].

#### 3.3.3. Obesity-Related Liver Diseases

Obesity-related liver diseases include liver steatosis, metabolic dysfunction, and non-alcoholic fatty liver disease (NAFLD).

MaR1 (2 μg/kg; *i.p.*) decreased lipogenic enzymes and liver triglycerides content while inducing fatty acid oxidation genes and autophagy and reducing fatty liver [29]. Additionally, MaR1 (35 μg/kg; *i.p.*) suppressed ER stress via AMP-activated protein kinase (AMPK)/sarcoendoplasmic reticulum Ca^2+^-ATPase 2b (SERCA2b), ameliorating obesity-related liver steatosis [30].

Injury to hepatocyte mitochondria is common in metabolic-dysfunction-associated fatty liver disease. MaR1 has optimal hepatic mitochondrial and metabolic efficiency, rescuing hepatocytes from mitochondrial dysfunction induced by obesogenic and fibrogenic insults [103].

NAFLD is a common metabolic liver disease. Decreased MaR1 might be involved in the development of NAFLD, which is proved by the lower MaR1 levels in NAFLD patients compared with those in non-NAFLD subjects (63.6 [59.9–73.9] vs. 73.1 [65.1–84.5] pg/mL) [104]. MaR1 can protect hepatocytes against lipotoxicity-induced apoptosis by activating the unfolded protein response (UPR) prosurvival mechanisms and opposing endoplasmic reticulum (ER) stress in liver cells, attenuating NAFLD [105].

#### 3.3.4. Non-Alcoholic Steatohepatitis and Liver Ischemia-Reperfusion Injury

Retinoic acid-related orphan receptor α (RORα) increased the transcriptional activity of 12-LOX and thereby increased MaR1, which can enhance the RORα ability to activate the M2 polarity of liver macrophages. Therefore, MaR1 can protect the liver from non-alcoholic steatohepatitis [31].

Hepatic ischemia/reperfusion (I/R) injury can be a major complication following liver surgery contributing to post-operative liver dysfunction. MaR1 (4 ng/g; *i.p.*) alleviated IR liver injury, facilitated by the activation of hepatocyte cell division, increased IL-6 cytokine levels, nuclear localization of NRF-2, decreased NF-κB activity, and the activation of the N-formyl peptide receptor 2 (FPR2, also called ALXR)/Akt signaling pathway. MAI (mitotic index) activity of hepatocytes was characterized by an intense cell division with 3.7- and 5.25-fold increases in the MaR1-sham and MaR1-IR groups, respectively. Moreover, MaR1-IR showed an increase of 41% in cell division related to MaR1-sham livers. IL-6 was increased 1.4 times in the MaR1-IR group compared to IR groups, while it was 0.2 and 6 times less than the IR and MaR1-IR groups, respectively, in the MaR1-sham group. Furthermore, the increase in nuclear Nrf2 of the MaR1-IR group was more than 7-fold that of the control [32,101,106,107]. MaR1 also attenuated acute liver injury by ameliorating inflammation [33].

#### 3.3.5. Pancreatitis

MaR1 (0.1,0.5,1 μg; *i.p.*) decreased serum levels of amylase, lipase, and inflammatory cytokines such as TNF-α, IL-1β, and IL-6 by inhibiting the activity of NF-κB and oxidative stress while increasing pancreatic acinar cell apoptosis and reducing pancreatic inflammation [34,35].

In conclusion, MaR1 can repair tissue after damage and attenuate inflammatory diseases in the digestive system, including colitis, hepatitis, and other liver diseases. Table 1 is the related compilation of preclinical models of the digestive system.

### 3.4. Endocrine System

The endocrine system comprises internal glands that maintain the body’s homeostasis through regulatory hormonal cascades [108]. Studies on maresin’s effects on the endocrine system have focused on obesity and diabetes, both of which feature persistent inflammation and are related to brown and white adipose tissues and the liver, which regulate insulin sensitivity.

#### 3.4.1. Obesity

Obesity is a complex disease affecting large populations and involving adipose tissue and endocrine responses. The production of bioactive SPMs, including MaR1 and pro-inflammatory mediators in the visceral adipose tissue of obese patients, was found to be significantly imbalanced [109]. In diet-induced obese (DIO) mice, MaR1 (2 μg/kg, *i.p.*, 10 days) reduced subcutaneous depot weight (from 2.4 ± 0.0 to 1.9 ± 0.1 g), serum white adipose tissue (WAT)-secreted lectin (from 43.9 ± 2.5 to 34.7 ± 2.7 ng/mL), and fasting glucose (167.9 ± 12.3 to 145.9 ± 8.4 mg/dL), while insulin levels and homeostatic model assessment were decreased insignificantly. While insulin tolerance tests (ITT) were enhanced by MaR1 (2 μg/kg, *i.p.*, 20 days) in leptin-deficient *ob/ob* mice, *Glut-4* expression was increased, and *Dpp-4* expression was reduced in both models [36]. Similarly, MaR1 (50 μg/kg, oral gavage, 10 days) given to DIO mice lowered fasting glycemia and reduced ITT glucose levels by around one-third. However, insulin-induced AKT phosphorylation, which regulates glucose transporter type 4 (GLUT4) translocation, was only partially restored in the muscle and not in epididymal WAT. In lean mice with acute MaR1 treatment (50 μg/kg, *i.p.*, 3 h), Akt phosphorylation in skeletal muscle and epididymal WAT was improved [37]. Moreover, fibroblast growth factor (FGF) 21 expression, associated with the regulation of insulin sensitivity, was increased in the liver and decreased in epididymal WAT of DIO mice. Both effects were reversed by MaR1 (50 µg/kg, oral gavage, 10 days), and inhibition of FGF receptor components was counteracted [38].

#### 3.4.2. Cold-Induced Resolution of Inflammation

Cold exposure stimulates β3-adrenergic receptors of brown adipose tissue (BAT), producing 14-HDHA, MaR2, and MaR2 isomers, which then act on the liver via circulation. Treatments of MaR2 (5 μg/kg/day, *i.p.*, 28 days; 10 μg/kg/day, *i.p.*, 26 days) on DIO mice did not change body weight yet downregulated plasma TNF-α levels and liver pro-inflammatory gene expression, possibly via the upregulation of triggering receptor expressed on myeloid cells-2 (TREM2) in monocytes and Kupffer cells. Notably, manifestations of hepatic steatosis, such as liver weight, triglyceride levels, lipogenic gene expression, steatosis, and alanine transaminase (ALT) index, were not altered in the latter treatment, nor was inflammatory gene expression in BAT [39].

#### 3.4.3. Type 2 Diabetes Mellitus (T2DM)

Plasma maresin levels were significantly lower in T2DM patients, especially those with diabetic foot ulcers. Notably, plasma levels of maresin were negatively correlated to BMI, waist circumference, waist–hip ratio, triglyceride, fasting plasma glucose (PG), 2hPG, glycated hemoglobin (HbA1C), and homeostasis model assessment for insulin resistance (HOMA-IR). On the other hand, its level positively correlated with acute insulin response, the first phase (0–10 min) insulin secretion, and homeostasis model assessment for beta-cell function (HOMA-β) [110]. Between tuberculosis patients with or without diabetes, the DHA-MaR metabolome group showed different network profiles with other lipid metabolomes, yet MCTR3 is one of the most abundant SPMs in the serum in both groups [111].

#### 3.4.4. Obesity and T2DM Treatments Related to Maresin

Weight loss and red wine failed to alter MaR1 levels released by patients with metabolic disease and T2DM, respectively. At the same time, one-anastomosis gastric bypass increased serum MaR1 in obese patients, which was lower than lean controls before intervention [112,113,114]. In another study on bariatric surgery, serum MaR1 levels had no significant difference between morbidly obese non-diabetic and diabetic patients. In contrast, both groups had higher 14-hydroxy docosahexaenoic acid (14-HDHA) than mildly obese non-diabetic controls. Interestingly, after surgery, serum MaR1 levels in morbidly obese non-diabetic subjects were significantly reduced (from 30.0 to 0 pg/mL) compared to their diabetic counterparts (9.5 to 8.8 pg/mL). Within the diabetic group after surgery, diabetic remitters exhibited a 0% change in MaR1 and reduced 58.2% of 14-HDHA; in non-remitters, MaR1 decreased by 36.9%, and 14-HDHA increased by 875.7%, reflecting the complexity of using its pathway metabolite14-HDHA as a sole biomarker [115].

Collectively, MaR1 successfully counteracted effects induced by obesity and T2DM, not only in the biochemical hierarchy but also in clinical manifestations, including lowering glucose levels, elevating insulin secretion, and resolving liver inflammation. Table 1 is the related compilation of preclinical models of the endocrine system.

### 3.5. Immune/Lymphatic System

The immune system serves as the defense system against external and internal noxious stimuli. Innate immunity acts through inflammation and phagocytosis against bacterial infection and trauma, which can lead to sepsis if uncontrolled. Adaptive immunity involves lymphocytes and lymphatic organs that target specific pathogens and monitors immune pathways comprehensively via the inhibition and expression of biomarkers. Though immune responses are developed to eliminate unwanted stimuli, hyperreactive host defenses can cause more tissue damage.

#### 3.5.1. Phagocytosis and Peritonitis

During *E. coli* self-limited peritonitis, MaR1 and its derivatives were identified in peritoneal exudates and distal spleens, while a panel of MCTR3, protein conjugates in tissue regeneration 3 (PCTR3), and REST Corepressor 3 (RCTR3) (50 ng each, *i.p.*) reduced PMN numbers in the exudate by ~70% and TNF-α by ~50% in mice with *E. coli* infection. Planaria regeneration after surgical injury reached almost 1.5-fold (CTR/vehicle) in MCTR3 (10 nM) incubation; in vitro, pretreatment of MCTR3 (10 nM) with human macrophages enhanced phagocytosis of *E. coli* by ~50% [40,116]. Similarly, MaR1 at 0.01 nM led to ~90% *E. coli* phagocytosis of primary human macrophages; parallel effects were seen in 22-OH-MaR1 (1 pM) and 14-oxo-MaR1 (1 pM) by ~75% and ~25%, though 22-OH-MaR1 was less potent than MaR1 at higher doses [41]. In toll-like receptor (TLR4)-engaged primary human monocytes, LPS-induced release of TNF, IL-8, IL-1β, and IL-12p40 was reduced by 50% in MaR1 pretreatment groups with corresponding MaR1 concentrations ranging from 1.0–3.0µm, while IL-10 production nearly doubled with MaR1 (1 μM) [42]. Similarly, exogenous MaR1 (100 nM) given after pretreatment of LPS in RAW264.7 murine macrophages reduced COX-2 mRNA levels by ~16% and IL-1β by ~5% [117]. Interestingly, MaR1 was produced to a higher extent in peritonitis induced by thioglycollate rather than zymosan, showing a tendency for thioglycollate to activate lipoxygenase pathways via DHA [118].

#### 3.5.2. Bacterial Infection and Sepsis

In an infection model, MaR1 (150 nM) counteracted the effects of *M. tuberculosis* (MOI = 5) in human-monocyte-derived macrophages, lowering intracellular bacterial burden from 7.5 to 4.8 × 10^4^ CFU/mL and TNF-α (from ~250 pg/mL to <50 pg/mL). A 1.66-fold change in bactericidal/permeability-increasing protein (BPI) expression was also induced by MaR1 compared to untreated cells [43]. Interestingly, LOX-mediated formation of SPMs, including MaR1, was only induced by pathogenic *Escherichia coli* (serotype O6:K2:H1) and *Staphylococcus aureus* but not non-pathogenic or attenuated *E. coli* [119]. In murine sterile peritonitis, Ω-3^+^ lipid emulsions increased levels of lipoxin A4 (LXA_4_), MaR1, and protectin DX (PDX). The emulsions also elevated survival rates of CLP-treated mice up to 60% and maintained temperature-reduced weight loss in mice with polymicrobial sepsis [120]. In a sepsis model simulating endotoxin-induced inflammation, MaR1 (optimal: 100 nM) reduced around half of TNF-α, IL-6, IL-1β mRNA, and protein levels in LPS-induced RAW264.7 cells and primary human peripheral blood mononuclear cells via the SIRT1/PGC-1α/PPAR-γ pathway [44].

#### 3.5.3. T Cell Differentiation and Lymphatic Obstruction

MaR1 (10 nM) downregulated cytokines produced by CD8^+^ and CD4^+^ T cells and reduced IL-2 production by around half in anti-CD3/CD28-stimulated T cells without decreasing T cell proliferation or expression of inhibitory receptors. in particular, MaR1 inhibited the differentiation of naïve CD4^+^ cells into Th1 or Th17 cells by downregulating T_bet_ and RORc while favoring differentiation into Treg by upregulating the expression of Foxp3^+^ (by ~30%) and CTLA-4 (by ~5%), and the production of IL-10 (by ~50%) [45]. Surgery-induced lymphatic obstruction (SLO) in the ileocecal region of African green monkeys showed low levels of endogenous SPMs on day 7 post-surgery, yet cumulative levels of D-series resolvins, lipoxins, and maresins combined increased on day 21 and 61 in the ileum and colon, paralleled with a resolution of inflammation that manifested on day 7, including thickened, shortened ileocecal mesentery covered by fibrin, edematous bowel loops and hyperplasia of stiffened lymph node [121].

Collectively, maresins regulated phenotypes of immune cells to reduce damage and promote repair as well as enhanced bacterial phagocytosis. Maresins also reduced cytokine release, striking a balance between controlled inflammation for a defense that limits self-damage. Table 1 is the related compilation of preclinical models of the immune and lymphatic system.

### 3.6. Integumentary/Exocrine System

The integumentary system consists of the skin, the body’s largest organ, and associated tissues, including the epidermis, dermis, and hypodermis, where subcutaneous adipose and connective tissue are located. The conjunctiva is a mucous layer connecting the inner surface of the eyelid to the anterior surface of the eyeball, which serves as an anatomical and immune barrier against external stimuli [122]. As the integumentary system is in constant contact with the external world, integrity must be maintained to protect the inner body.

#### 3.6.1. Skin Inflammation, Psoriasis, Melanoma

Skin diseases can range from inflammation and edema to more severe forms such as psoriasis and melanoma. In a mouse UVB-induced skin inflammation model, MaR1 (10 ng/mouse, *i.p.*, 10 min before UVB irradiation) reduced skin edema, manifested by a decrease of ~5 mg skin weight from the edema-induced increase of ~8 mg. Other UVB-induced effects downregulated by MaR1 include neutrophil recruitment (by ~60%), keratinocyte apoptosis (by ~60%), epidermal thickness (by ~50%), matrix metallopeptidase 9 (MMP-9) activity (by ~66%), and collagen degradation (saving ~40% of skin collagen from UVB-induced 80% loss), via the suppression of oxidative stress [46]. Topical MaR1 (100 ng in 20 μg ethanol/ear) given before imiquimod (5 d) or Interleukin 23 (IL-23, 16 d) administration in mouse psoriasis models ameliorated ear swelling by about 40–50% and reduced epithelial thickness by ~33~50%, dermal edema, and several CD45^+^ cells and Ly-6G^+^ cells, via inhibition of the IL-23/IL-17 axis [47]. Moreover, in transgenic mice with melanoma xenografts, endogenously produced n-3 polyunsaturated fatty acids (PUFAs) might have slowed the tumor growth rate in part through the production of anti-inflammatory mediators, including maresins [123].

#### 3.6.2. Ocular Surface Inflammatory Disease

Mucin and tears are essential protective components of the ocular surface. Physiologically, SPMs at bioactive concentrations were detected in human emotional tears, though MaR1 and MaR2 were absent [124]. In contrast, exogenous maresins increased high molecular weight glycoprotein (HMWG) secretion in primary rat conjunctival goblet cells by 2.4 ± 0.4-fold (MaR1, optimal: 10^−9^ M) and 2.2 ± 0.3-fold (MaR2, optimal: 10^−8^ M) above basal and significantly promoted [Ca^2+^]_i_ production, with optimal changes in peak [Ca^2+^]_i_ at 220.3 ± 37.8 nM (10^−8^ M MaR1) and 189.2 ± 14.5 nM (10^−8^ M MaR2), via different but overlapping pathways. MaR1 and MaR2 were also able to counteract the overproduction of mucin, a manifestation of ocular allergy, by reducing the histamine (10^−5^ M)-induced increase in HMWG secretion, from 2.4 ± 0.3- to 0.87 ± 0.21-fold (MaR1) and 1 ± 0.13- to 1.1 ± 0.2-fold (MaR2) above basal; the change in [Ca^2+^]_i_ production was also reduced from 265.0 ± 45.6 to 84. 0 ± 18.8 nM (MaR1) and 269.5 ± 28.9 to 150.67 ± 50.78 nM (MaR2). These results show the potential for MaR1 and MaR2 to develop as different therapeutic agents to counteract eye dryness and allergic conjunctivitis [125,126].

#### 3.6.3. Adipose Tissue

The adipose tissue not only protects organs and provides energy but also secretes a variety of lipid mediators. To understand the correlation of SPM profiles and brown adipose tissue (BAT) dysfunction in aging, obesogenic mice, it was found that maresins were the second most abundant LMs in young chow diet mice and one of the main contributors to differences between age and diet groups. Interestingly, almost all maresin derivative levels in BAT of aged mice fed with the high-fat diet, but with 15% replacement of dietary lipids to DHA-rich fish oil concentrate, were more than 3-fold compared to other groups, including young chow diet mice [127]. Moreover, in a TNF-α-induced lipolysis cell model, an abnormal increase in glycerol secretion in 3T3-L1 adipocytes was totally counteracted by MaR1 (100 nM, at 48 h). Levels of hormone-sensitive lipase (HSL) and perilipin were also reduced by TNF-α to 30% (HSL) and 45% (perilipin) of basal levels. Still, they were restored by MaR1 (100 nM, at 48 h) to 50% (HSL) and 75% (perilipin). Meanwhile, MaR1 (100 nM, at 48 h) showed its ability to prevent TNF-α-induced autophagy by restoring ~25% of p62 protein expression and returning the LC3II/LC3I ratio to the basal level, though cell viability was not altered [128]. Similarly, 24 h co-treatment of MaR1 (10 nM) with TNF-α (10 ng/mL) in primary human adipocytes reversed TNF-α-induced chemerin gene expression and protein secretion back to the basal level [48]. In short, maresins were replenished by DHA supplementation in vivo and inhibited inflammation in in vitro models.

Collectively, maresins counteracted external stimuli in the skin, maintained tear film homeostasis in the conjunctiva, prevented histamine-induced mucin overproduction, and regulated inflammation responses in the adipose tissue, counteracting adverse effects of inflammation. Table 1 is the related compilation of preclinical models of the integumentary and exocrine systems.

### 3.7. Musculoskeletal System

The musculoskeletal system includes bones, muscles, cartilage, ligaments, tendons, and connective tissues, supporting body weight. Arthritis, as an important disease of the musculoskeletal system, is the swelling and tenderness of one or more joints. The main symptoms of arthritis are joint pain and stiffness, and the most common types of arthritis are osteoarthritis and rheumatoid arthritis. The research investigates if maresin can be useful for musculoskeletal diseases such as arthritis, Achilles tendinopathy, or muscle dysfunction.

#### 3.7.1. Achilles Tendinopathy and Bone/Muscle Regeneration

In tendon-derived stromal cells from patients with Achilles tendinopathy or Achilles rupture, MaR1 (10 nM) regulated the pro-inflammatory phenotype and promoted resolution, countering inflammation [129].

MaR1 with pro-regenerative functions could restore acute inflammation and promote the mesenchymal stroma cells’ proliferation and migration, osteogenesis, and angiogenesis by upregulating the expression of 12-LOX, suggesting the function of inflammation resolution, bone regeneration, and muscular injuries associated with aging [130,131]. MaR1 is a leucine-rich repeat-containing G-protein-coupled receptor 6 (LGR6) ligand, which is necessary for normal osteogenesis. The percentage of MaR1 (100 ng/mL)-stimulated LGR6-mediated cAMP activity was about three times that of the control group [132]. In a mouse model (underwent tibial fracture) experiment, MaR1 (5 µg/kg, *i.p.*) decreased the percentage of pro-inflammatory macrophages (Veh, 20.5 ± 1.7; MaR1 9.8% ± 4.3) and serum levels of inflammatory cytokines IL-6 (Veh, 108.6 ± 29.8; MaR1, 39.4 ± 15.8 pg/mL), IL-10 (Veh, 56.8 ± 16.3; MaR1, 27.2 ± 4.8 pg/mL), and TNFα (Veh, 33.4 ± 4.1; MaR1, 13.2 ± 3.0 pg/mL). MaR1 treatment also increased the bone volume (BV) within the fracture callus (Veh, 4.0 ± 1.8; MaR1, 5.5 ± 1.4 mm^3^) and the relative amount of bone within the fracture callus (BV/TV—Veh, 0.5 ± 0.2; MaR1, 0.7± 0.2). Bone content was higher in MaR1-treated samples (Veh, 35.3 ± 5.2; MaR1, 45.1 ± 4.2%) [49].

#### 3.7.2. Arthritis

Research insights into osteoarthritis. In equine synoviocytes, the osteoarthritis mediator gene expression osaR1 (200 nM) group was significantly increased compared to the control group [133]. MaR1 suppressed IL-1B-induced rat fibroblast-like synoviocytes and MMP13 secretion by activating the P13k/Akt pathway while inhibiting the NF-κB pathway [134].

Rheumatoid arthritis (RA) is an autoimmune disease that may damage a wide variety of body systems, including the skin, eyes, lungs, heart, and blood vessels. Compared with controls, the MaR1 concentration was higher in patients with inactive RA and lower in patients with active RA. Expression of the Treg transcription factor FoxP3 was the highest in inactive RA and the lowest in active RA, while the Th17 transcription factor RORc showed a reverse trend in the collagen-induced arthritis mouse model. The intervention of MaR1 (0, 20, and 100 ng, *i.v.*) improved the imbalanced Treg/Th17 ratio. MaR1 increased the Treg cell portion while reducing the Th17 cell proportion dose dependently. Furthermore, miR-21 was verified as MaR1 downstream mRNA, which was upregulated by MaR1, modulating the Treg/Th17 balance and thus ameliorating the RA progression [50]. MCTR3 potently decreased joint inflammation and promoted tissue repair. MCTR3 was mediated via the reprogramming of circulating monocytes to yield macrophages, upregulating arginase-1 to enhance the pro-resolving inflammation and tissue repair function [135].

In summary, maresin is proven to have a significant effect on inflammation resolution and tissue regeneration, helping to ameliorate inflammatory diseases such as arthritis or musculoskeletal injuries. It may be a potential therapy for inflammatory disease in the future. Table 1 is the related compilation of preclinical models of the musculoskeletal system.

### 3.8. Nervous System

The nervous system regulates perception, cognition, and responsiveness to stimuli. Neuroinflammation could lead to cognitive disorders, cerebrovascular diseases, other dysfunctions, and chronic pain.

#### 3.8.1. Cognitive Disorders

Alzheimer’s disease (AD) is a multifactorial inflammatory disease causing cognitive decline. In mice receiving bilateral hippocampal Aβ injection, MaR1 (0.01 µg, *i.c.v.*)-treated mice were faster in the Morris Water Maze (MWM) [51]. Intranasal delivery of the SPM-combined solution (RvE1, RvD1, RvD2, MaR1, and NPD1, 40 ng per LM) given to *App^NL-G-F/NL-G-F^* mice thrice a week for 9 weeks reduced microgliosis and recovered 57% of gamma oscillation power [52]. Furthermore, in humans, MaR1 of cerebrospinal fluid was lower in the mild cognitive impairment group compared to their subjective counterparts but was negatively correlated with the mini-mental examination score (MMSE); in the entorhinal cortex, MaR1 was higher in AD patients [136,137]. MaR1 was lower in mice with the APOE4 genotype compared to APOE3 and higher in females and enhanced Aβ phagocytosis in vitro [138,139]. In another form of dementia, frontotemporal dementia carriers with C9orf72 expansion had higher maresin levels than those with other mutations [140].

Surgery could also induce cognitive impairment. In C57BL/6 and Ccr2^RFP/+^Cx3cr1^GFP/+^ mice undergoing orthopedic surgery, MaR1 (100 ng, *i.p.*) prophylaxis before fracture reversed surgery-reduced freezing time by ~20% in contextual fear conditioning [53]. Similarly, MaR1 (10 nM, *i.p.*, 3 days) pretreatment to rats improved MWM performances after sevoflurane exposure, showing the ability of MaR1 to enhance memory [54].

#### 3.8.2. Cerebrovascular Diseases

Ischemia in the brain impairs the delivery and utilization of oxygen and nutrients, leading to cerebral dysfunction. MaR1 (0.05 μg, intrathecal) decreased escape latency in MWM by 20~30 s maximally and alleviated the blood–brain barrier, BBB) changes in rats with chronic cerebral hypoperfusion [55]. In mice with ischemia/reperfusion injury, MaR1 (1 ng, intracerebroventricular) decreased ~40% of original infarct volume and reduced brain damage via modulating silent information regulator 1 (SIRT1) signaling [56,141]. Regarding stroke, patient plasma MaR1 showed no correlation with the MMSE score within 7 days of stroke onset, while another study reported negative correlations with the California Verbal Learning Test on the 7th day and 6-month follow-up, suggesting that MaR1’s impacts are sophisticated [142,143].

#### 3.8.3. Multiple Sclerosis (MS), Spinal Cord Injury (SCI), and Amyotrophic Lateral Sclerosis (ALS)

Unresolved inflammation in MS patients and mice with experimental autoimmune encephalomyelitis (EAE) might come from the inability to synthesize SPMs. Daily MaR1 injections (1 μg, *i.p.*, 21 d) in EAE mice resulted in lower average EAE scores (~2.5) than the vehicle (~4.2) and a decreased area of myelin loss [57]. In SCI mice, MaR1 (1 μg, *i.v.*, 7 d) raised Basso Mouse Scale scores (4.6 ± 0.2) compared to controls (3.5 ± 0.2) on day 28 post-injury as well as improved gait symmetry and stance/width stepping variability scores, by elevating myelinated axons by ~20% [58]. Similarly, MaR1 (1 mg/kg, *i.p.*, 11–13 d) given to mice with spinal muscle atrophy decreased righting reflex latency by ~40 s and negative geotaxis test latency by ~150 s, improving motor functions [59].

#### 3.8.4. Pain

Inflammatory pain is characterized by hypersensitivity, while neuropathic pain results from neural anomalies. MaR1 pre- and post-treatments (optimal: 10 ng, *i.t.*) alleviated acute and chronic inflammatory pain in mice, reducing the difference in the withdrawal threshold between the baseline (at zero-time) and after 1–5 h carrageenan stimulation in mechanical (by max: ~2 g) and thermal hyperalgesia (by max: ~5 s); MaR1 pretreatment (10 ng, *i.t.*) also reduced flinches and time spent licking the paw by ~50%, mitigating overt pain [60]. Similarly, MaR2 (optimal: 30 ng, *i.p.*) reduced LPS-induced mechanical and thermal hyperalgesia, lowering Δreaction by 2~4 g and increasing latency by 5~10 s; the injured/non-injured paw weight ratio was also increased from ~0.6 to ~0.8 by MaR2 [61]. Neuropathic pain was also attenuated in rats by MaR1 (100 ng/10 µL, *i.t.*) after spinal nerve ligation, raising the ipsilateral mechanical withdrawal threshold by ~10 g and thermal paw withdrawal by latency by ~10 s [62]. Parallel results were obtained from MaR1 (optimal: 100 ng, *i.t.*, 3 d) given to rats with non-compressive lumbar disc herniation having radicular pain, which is associated with NLR family pyrin domain containing 3 (NLRP3) inflammasome and caspase 1 activation [63,64].

In mice with peripheral nerve (sciatic nerve crush) injury, MaR1 (500 ng, applied onto damaged nerves using hemostatic gelatin sponge) reduced gastrocnemius atrophy and performed better than nerve growth factor (NGF) in the rotarod, von Frey, and Hargreaves tests, improving motor and sensory functions. MaR1 (optimal: 100 ng, *i.t.*) mitigated neuropathic pain and (50 ng, intraplantar) maintained pain threshold, unlike NGF [65]. Furthermore, MaR1 used as peri- (500 ng, *i.v.*) and post-operative treatments (500 ng, *i.t.*) showed the most potent effects by inhibiting fracture-associated post-operative pain in mice compared to DHA and other SPMs [66]. Lastly, K/BxN-induced mechanical hypersensitivity without joint swelling in rheumatoid arthritis was ameliorated in mice treated with MaR1 (100 ng, *i.p.*), with a later onset and longer duration after repeated injections [67].

As therapeutic candidates, maresins exerted neuroprotective and analgesic effects without cytotoxicity or hypersensitivity. Table 1 is the related compilation of preclinical models of the nervous system.

### 3.9. Reproductive System

Both male and female reproductive systems contain external organs and internal organs. The major function of the reproductive system is to ensure the survival of the species. The reproductive system produces ovum and sperm cells, transports and sustains these cells, nurtures the developing offspring, and produces hormones.

#### 3.9.1. Human Milk

Human milk contains nutrients and bioactive products relevant to infant development and immunological protection. It possesses specialized pro-resolving mediators, including maresins, that enhance human macrophage efferocytosis and bacterial containment. The MaR1 level in healthy human milk (4–8 weeks post-partum) is 20.8 ± 6.3 (pg/mL). Treating MaR1 (50 ng, *i.p.*) in a mouse peritonitis model shortens the resolution interval and magnitude of PMN infiltration by 76% and 58%, respectively [68].

#### 3.9.2. Localized Provoked Vulvodynia (LPV)

Localized provoked vulvodynia (LPV) is the most common cause of chronic dyspareunia in premenopausal women, characterized by pain with a light touch to the vulvar vestibule surrounding the vaginal opening. The devastating impact of LPV includes sexual dysfunction, infertility, depression, and even suicide. In LPV, the vestibule expresses a unique inflammatory profile with elevated levels of pro-nociceptive pro-inflammatory mediator prostaglandin E2 (PGE2) and interleukin-6 (IL-6), which are linked to lower mechanical sensitivity thresholds. In a murine vulvar pain model, topical treatment with the SPM, maresin 1, decreased sensitivity by increasing the pain threshold and suppressed PGE2 levels. Docosahexaenoic acid (DHA), a precursor of maresin 1, was also effective in reducing PGE2 in vulvar fibroblasts and rapidly restored mouse sensitivity thresholds. Overall, SPMs and their precursors may be safe and efficacious for LPV [69].

#### 3.9.3. Polycystic Ovary Syndrome

Polycystic ovary syndrome (PCOS) is an endocrinological disorder that affects 5–15% of women of reproductive age and is a frequent cause of infertility. Major symptoms include hyperandrogenism, ovulatory dysfunction, and often obesity and/or insulin resistance. PCOS also represents a state of chronic low-grade inflammation that is closely interlinked with metabolic features. MaR1 and MaR2 serum levels in the PCOS group were 81.8 and 231.0 pg/mL, respectively, while in the healthy group they were 21.5 and 93.0 pg/mL, respectively. The ratio (sum of pro-inflammatory molecules)/(sum of SPMs plus hydroxylated intermediates) reflecting the inflammatory state was significantly lower in the group of healthy women. In conclusion, there is a strong pro-inflammatory state in PCOS patients. Further research may clarify whether supplementation with DHA may improve this state [144].

#### 3.9.4. Pre-Eclampsia (PE)

Recent data indicated an imbalance of leukotriene B4 (LTB4) and MaR1 levels in pre-eclampsia (PE). Research showed that plasma concentrations of LTB4 were higher in PE women than in normotensive pregnant women, who presented higher levels of LTB4 than non-pregnant women. In addition, PE women had a decreased MaR1/LTB4 ratio. The findings suggested that the PE state resulted in the systemic overproduction of LTB4 in relation to MaR1, and it may be involved with disease pathogenesis [145].

In conclusion, maresin has been proven to reduce tissue inflammation and damage. The relation between maresin levels and the above diseases shows that maresin may be one of the potential therapeutics in the female reproductive system. Table 1 is the related compilation of preclinical models of the reproductive system.

### 3.10. Respiratory System

The respiratory system is a network of organs and tissue that helps us breathe, including the airway, lungs, and vessels. Some conditions, such as viruses or bacterial infections, can cause inflammation in the respiratory system. MaR1 has been proven to suppress inflammation and the production of inflammatory cytokines while increasing anti-inflammatory cytokines.

#### 3.10.1. Acute Respiratory Distress Syndrome (ARDS)

Acute respiratory distress syndrome (ARDS) is a devastating condition of acute lung inflammation. Patients with ventilation for fewer than 7 days showed higher MaR1 values than those who were ventilated for more than 14 days. In addition, MaR1 concentrations were also increased in patients who stayed in ICU for fewer than 7 days, compared with those who stayed longer than 14 days [146]. MaR1 was significantly increased during platelet–neutrophil interactions, restraining neutrophil-platelet aggregates (N-PAs) and acute pulmonary inflammation to restore homeostasis of the injured lung [147].

#### 3.10.2. Virus and Bacterial Infection: COVID-19 and Bacterial Pneumonia

Plasma from COVID-19 patients presented higher amounts of pro-inflammatory mediators compared to the healthy group (65.7 vs. 10.2 pg/mL, respectively), and the ratio between total plasma pro-inflammatory mediators versus total SPMs was 13.2 to 0.4 times, respectively [148]. Secondary bacterial pneumonia is a common complication of Influenza A virus (IAV) infection, leading to excess morbidity and mortality. Research showed that MCTRs increased macrophage resilience mechanisms after IAV to protect against secondary infection from Streptococcus pneumoniae [149].

#### 3.10.3. Chronic Rhinosinusitis and Asthma

Research showed that MCTRs are the most abundant cysteinyl lipid mediator and could attenuate airway contraction in human precision-cut lung slices [70]. MaR1 (10 ng, *i.v.*) suppressed the activation of the NF-κB signaling pathway, thus reducing COX-2 and ICAM-1, preventing inflammatory cell infiltration in the bronchoalveolar lavage fluid and excessive mucus production. Meanwhile, maresin inhibited the activation of the NLRP3 inflammasome, the Th2-type immune response, and oxidative stress [71,150].

#### 3.10.4. Acute Lung Injury (ALI)

Acute lung injury is characterized by lung inflammation and diffuse neutrophil infiltration, controlled by neutrophil apoptosis. The percentage of neutrophil apoptosis in the MaR1-treated group was about three times that of the control group. Maresin-1 accelerated the resolution of inflammation in lipopolysaccharide-induced acute lung injury (LPS-induced ALI) and reduced the production of pro-inflammatory cytokines while upregulating the production of IL-10 [151,152]. MaR1 alleviated LPS-induced ALI by protecting the endothelial glycocalyx, which limited the access of certain molecules to the cell membrane and upregulated the expression of the tight junction protein to decrease lung injury permeability [153,154]. In addition, MaR1 (2 ng/g in the murine model and the pulmonary epithelial cell line) inhibited cell death, inflammatory cytokine levels, and oxidative stress through the inactivation of the ALX Homeobox 1 (ALX)/PTEN-induced putative kinase 1 (PINK1)-mediated mitophagy pathway, protecting against LPS-induced ALI [72]. The alveolar fluid clearance (AFC) rates of LPS, LPS with maresin-1 treatment, and the control group were 10.1 ± 1.1,18.9 ± 2.1, 25.6 ± 2.9 (%), respectively. Maresin-1 significantly promoted AFC by upregulating the epithelial sodium channel and Na^+^-K^+^-adenosine triphosphatase expression in vivo [73].

#### 3.10.5. Airway Inflammation and Lung Fibrosis

Airway inflammation associated with acute and repetitive exposure to organic dust is a high-risk factor for chronic bronchitis and obstructive pulmonary disease. In organic dust exposure studies, MaR1 significantly decreased bronchoalveolar lavage neutrophil infiltration and intracellular adhesion molecule-1 (ICAM-1) expression [74]. MaR1 also reduced cytokines’ production such as IL-6, IL-8, and TNF-α of bronchial epithelial cells dose-dependently [155]. Lung fibrosis results from lung fibroblast migration, proliferation, and differentiation into a myofibroblast-like cell type induced by TGF-β. MaR1 protected tissue and reversed the epithelial-to-mesenchymal transition (EMT) phenotype by decreasing TGF-β, enhancing the survival rate (best dose response at 1 μg) [75,156,157].

In summary, MaR1 showed a significant effect of resolving inflammation and promoting tissue regeneration. The suppression of the inflammation and production of inflammatory/pro-inflammatory cytokines can be useful for lung inflammatory diseases, restoring lung tissue back to homeostasis. Table 1 is the related compilation of preclinical models of the respiratory system.

### 3.11. Urinary System

The urinary system’s function is to filter blood and create urine as a waste by-product. The organs of the urinary system include the kidneys, renal pelvis, ureters, bladder, and urethra.

#### 3.11.1. Cystitis

Inflammation is a central process in most benign bladder disorders, and the outcome is a delicate balance between initiating factors and resolving factors. Inflammation in tissue is associated with weight gain, most commonly from edema. In murine model research, it showed that SPMs, including MaR1 (25 μg/kg, *i.p.*), promoted epithelial wound/barrier repair, alleviated inflammation, and resulted in reduced bladder weights. The percentage of scratch closure of the MaR1 group was twice that of the control group. These results provided promising therapeutic targets for inflammatory bladder diseases [76].

#### 3.11.2. Diabetic Nephropathy (DN)

Diabetic nephropathy (DN) is a major complication of diabetes mellitus. MaR1 inhibited the NLRP3 inflammasome, TGF-β1, and fibronectin (FN) in mouse glomerular mesangial cells, suggesting that MaR1 may have a protective effect on DN by mitigating inflammation and early fibrosis [77].

#### 3.11.3. Sepsis-Associated Acute Kidney Injury (S-AKI)

Sepsis-associated acute kidney injury (S-AKI) is a common complication in hospitalized and critically ill patients, which increases the risk of multiple comorbidities and is associated with extremely high mortality.

The 7-day survival rates of the sham group, the cecal ligation and puncture (CLP) group, the MaR1 low dose (LD-MaR1, 0.5 ng) group, and the MaR1 high dose (HD-MaR1, 1 ng) group were 100%, 16.67%, 58.33%, and 75%, respectively. Research showed that MaR1 significantly increased the 7-day survival rate of septic mice and the anti-inflammatory factor while reducing bacterial load and pro-inflammatory cytokines. In addition, MaR1 dose-dependently reduced renal injury scores and serum creatinine and urea nitrogen levels in septic mice while inhibiting renal neutrophil infiltration and myeloperoxidase (MPO) activity [78]. In conclusion, MaR1 attenuated kidney inflammation by reducing neutrophil infiltration and inhibiting the NF-κB pathway activity, apoptosis, oxidative stress, and mitochondrial dysfunction to protect against septic AKI [158].

Ferroptosis is unique among different types of regulated cell death and closely related to organ injury, and nuclear factor-erythroid-2-related factor 2 (Nrf2) is crucial to the regulation of ferroptosis. Maresin conjugates in tissue regeneration 1 (MCTR1) inhibited ferroptosis in sepsis-associated acute kidney injury (SA-AKI) and elevated the expression of Nrf2. The percent survival of the CLP group was about twice that of the MCTR + CLP group. As a result, MCTR1 ameliorates multi-organ injury and improves survival in animal models of sepsis [79].

#### 3.11.4. Renal Ischemia/Reperfusion Injury

Inflammation and oxidative stress play a crucial role in the pathogenesis of renal ischemia/reperfusion injury (IRI). In a murine model, the histologic score of the IRI + MaR1 group (1.0 ng, *i.v.*) was about two-thirds that of the IRI group. Meanwhile, MaR1 remarkably mitigated renal IRI-induced inflammation and oxidative stress. The results indicated that MaR1 markedly protected against renal IRI by the reduction in histologic changes and renal dysfunction by inhibiting the TLR4/MAPK/NF-κB pathways, which mediate anti-inflammation, and by activating the Nrf2 pathway, which mediates antioxidation [80].

To sum up, MaR1 inhibits inflammation, which helps to increase the survival rate associated with diabetes and kidney disease. Its ability to ameliorate organ injury and histologic changes suggests that MaR1 may be a future therapeutic target for the above diseases. Table 1 is the related compilation of preclinical models of the urinary system.

### 3.12. Discussion

The relative relationship between the action timing of maresins and the onset of diseases may affect the degree of disease mitigation. Based on the experiments conducted to date, the concentrations of maresins administered are usually between micrograms and nanograms, and there should be a range of optimal concentrations for each situation, which is worthy of further study to obtain the best choice. The modes of administration are mostly by injection, and some are administered orally or with special carriers. In some experiments, additional dosages or adjustments of the duration of action are used to achieve the effect, but the subsequent mechanism of maresins is not yet known. The mechanism of maresins may be quite complex due to the intricacies of the human environment and the large number of immune cells and cytokines involved in the inflammatory response.

Preclinical studies on maresins related to human organ systems have reported their efficacy and potential in treating different inflammatory diseases and conditions. There are many other mechanistic studies that have also supported their therapeutic action and are worth discussing. The following is additional significant information about maresins as biomarkers, interactions in platelet aggregation, supplementation, and other resolving actions.

#### 3.12.1. MaR1 in Human Tissue Fluid and as a Biomarker

Although maresin 1 was first found in self-limiting peritonitis exudate from mice, it was soon discovered that human macrophage also produces MaR1 and presents in human serum [159]. MaR1 was also found in several sources of human tissue fluid and could potentially serve as a biomarker. Saliva was found to have a greater amount of MaR1 in periodontitis patients (0.69 vs. 0.49 ng/mL in control), and patients with cardiovascular disease (CVD) and generalized periodontitis had a more pronounced level. However, within the observed period in the study, no correlation was found with the rate of periodontitis progression, but evidence suggested that an increase in MaR1 and a reduction in protectins’ (PD) salivary levels could be predictors of developing CVD and/or periodontal disease [160,161]. Moreover, it found that MaR1, 14-HDHA, and 7(s)-MaR1 have correlations with different subgingival microbiota of subgingival plaque between the healthy group, periodontitis before scaling and root planning (SRP) treatment, and periodontitis after SRP treatment [162]. In addition, serum from patients with post-menopausal osteoporosis (PMOP) (124.68 ± 31.35 pg/mL) and osteopenia (140.1 ± 30.5 pg/mL) are different from the healthy group (167.4 ± 24.9 pg/mL). Serum MaR1 levels were positively correlated with femoral neck, lumbar spine, and hip bone mineral density. The results suggest that MaR1 may serve as a new non-invasive diagnostic biomarker for PMOP [163]. Moreover, the urine level of MaR1 is significantly lower (about 50%) in subjects with stage 3–4 nephropathy than in healthy subjects and those with stage 1–2 nephropathy. In patients with T2DM (with or without kidney disease), MaR1 serum concentration is lower (about 50%) compared to healthy controls, and the diabetic kidney disease (DKD) group had the lowest concentration. Additionally, MaR1 concentration was correlated with high-density lipoprotein cholesterol (HDL-C) and the estimated glomerular filtration rate (eGFR). In conclusion, a decreased serum MaR1 level was correlated with the development of DN and DKD, which could be an indicator [77,164,165]. MaR1 is also present in joint synovial fluid. Regarding joint-related diseases, DMARDs are used to treat patients with RA [166,167]. In order to predict the effectiveness of disease-modifying antirheumatic drugs (DMARDs), the research found that the plasma concentration of maresin, as a biomarker, is predictive of DMARD responsiveness and RA pathotypes [168]. In the plasma of amyotrophic lateral sclerosis (ALS) patients, a reduction in 14-HDHA was found, demonstrating an imbalance in the resolution process of inflammation [169]. In addition, C-reactive protein (CRP), which is an inflammation protein marker produced by the liver in the plasma, was associated with EPA and DHA levels dose dependently [170].

#### 3.12.2. Platelets’ Aggregation and Supplementation

Maresin 1, as one of the specialized pro-resolving mediators (SPM), was significantly increased during platelet–neutrophil interactions, restraining neutrophil-platelet aggregates (N-PAs) and acute pulmonary inflammation from restoring homeostasis of the injured lung [147]. Aggregation is a problem during cold storage of apheresis platelets, and SPM treatment (MaR1: 100 nM) reduced the activation and preserved the functions of platelets at seven days [171]. 14S-HpDHA (≥1 μM), the precursor of maresin, also reduced the aggregation of platelets by 90%, implying that the intake of DHA may be beneficial in treatment [5]. However, in terms of the supplementation of n-3 fatty acids, whether it increases the concentration of endogenous maresin-associated products in the blood or tissue when fighting against bacteria, in the model of Alzheimer’s disease, or during human pregnancy in different stages, still needs further investigation [172,173,174,175,176]. Ingestion of fish oil can also upregulate peripheral blood MaR2 and MaR2n-3 DPA levels (4.5 g total fatty acids, 2–4 h), and the concentration of MaR1n-3 DPA (4.5 g total fatty acids) was negatively correlated with the expression of monocyte activation marker CD49d [177]. For patients with obesity, after consuming 2 g/d of marine oil for about one month, the plasma level of MaR1 increased 4.7-fold, and B cell IgG decreased [178]. In short, MaR1 may have the ability to prevent the aggregation of platelet storage, and daily ingestion of fish oil might modulate the functions of inflammatory cells.

#### 3.12.3. Other Modulating Functions and Cancer Cell Suppressibility

MaR1 production from M2 macrophages could be enhanced by PMN microparticles and phagocytosis of apoptotic PMNs, while the MaR1 level was lower in zymosan-induced peritonitis when vacuolar-ATPase was blocked. Neogenin1 deficiency is also associated with an increase in exudate MaR1 and enhanced macrophage phagocytosis via 12/15 LOX activity [159,179,180]. In contrast, the heme oxygenase-1 (HO-1)/carbon monoxide (CO) pathway interacts with SPMs in a positive feedback manner, with macrophage phagocytosis of zymosan reaching its maximal potential when CO and MaR1 (0.01 nM) were concomitantly present. An MaR1 (0.1 nM)-induced increase (30–40%) in phagocytosis was also abrogated by HO-1 inhibition [181]. In microRNA (miRNA)-4661-transfected mice with zymosan-induced peritonitis, biosynthesis of SPMs, including MaR1, was increased, yet MaR1 (10 nM) reduced miR-4661 expression in vehicle- and zymosan-treated primary human macrophages and neutrophils. MaR1 (10 nM) also reversed the upregulation of activator protein (AP)-1 and NF-κB1 signaling induced by zymosan, but effects on miR-4661 downstream pathways were inhibited by pertussis toxin [182]. For physiological hypoxia, M2 macrophage efferocytosis of apoptotic PMNs and senescent erythrocytes was enhanced up to 10-fold via upregulation of endogenous SPM levels, including MaR1 and MaR2, when these cells were co-incubated in hypoxic environments, which could mimic inflammatory sites, bone marrow, and the spleen. As neutrophils depend mostly on glycolysis, hypoxia elevated neutrophil SPM production by ~5-fold and MaR1 by ~3-fold, thus ameliorating neutrophil-induced inflammation [183]. Moreover, MaR1 (10–100μM) exhibited suppressibility of breast, pancreatic, and colon cancer cells’ growth to different extents (range from 62 to 92%), suggesting that there might be an optimal ω-3 fatty acids choice to inhibit the growth of each cancer cell [184]. The main limitation of this system-based systematic review is that most of the studies are preclinical studies, but it is also the focus of this review to synthesize all the translational information from animal studies, which is extremely critical. Compared with systematic human reviews, data extractions can be more challenging because most of the literature does not contain raw data or detailed numeric information in paragraphs but the data are presented as graphs. Therefore, many of the values presented in the table are presented with relative ratios calculated from the original figures. Furthermore, this article is written in terms of organ systems and diseases so that we can clearly see which diseases or conditions are being studied. However, the reference value of cell experiments, animal experiments, and human experiments are not identical and should be categorized more carefully to eliminate confusion.

Despite the fact that current published studies on maresins are mostly in the preclinical stage, their huge potential applications in each organ system are well-demonstrated. Future translational studies and human trials are expected and warranted. Moreover, further investigations into the pharmacokinetics, bioavailability, and safety profiles are necessary to build their potential for clinical translation.

## 4. Conclusions

Maresins have demonstrated their competence to resolve inflammation for organ protection and tissue regeneration in various diseases and systems. The nervous system has the highest number of studies, followed by the cardiovascular, digestive, and respiratory systems. In different animal disease models, various delivery methods and therapeutic dose responses have gradually been tested by different research groups. These integrated findings highlight the importance of studying maresins in various organ systems and experimental contexts. It underscores the need for further translational research to fully understand the therapeutic potential, mechanisms of action, and optimal dosing with delivery methods for clinical applications of maresin.

## Figures and Tables

**Table 1:** Maresin preclinical studies with delivery methods, optimal concentrations, and effects in the 11 human organ systems.

1.1 Oral, Dental, and Craniofacial System				
Preclinical Models	Administration of Maresins	(Optimal) Concentration	Efficacy	References
Patients with localized aggressive periodontitis (Stage IV Grade C molar-incisal pattern periodontitis)	MaR1, in vitro incubation, patient-derived neutrophils, and macrophages	1 nM	1. Enhanced phagocytosis (31 to 65% increase), restored bactericidal capacity (22 to 38% reduction in bacterial titers) and increased intracellular ROS generation (26 to 71% increase).	(Wang, C.W., 2015) [12]
Healthy human PDL cells, under the stimulation of *P. gingivalis* LPS	MaR1, in vitro incubation	10 nM	1. Increased healthy human PDL cell survival rate and autophagy, decreased its apoptosis and production (about 50% reduction) of inflammatory factors (IL-6, IL-8, TNF-α, and IL-1β).	(Du, L., 2018) [13]
Healthy human PDL cells, under the stimulation of IL-1β and TNF-α	MaR1 and/or RvE1, in vitro incubation	10 nM	1. Increased the expression of pluripotency, migration, viability, and differentiation of hPDLSCs into PDL-like cell phenotype (IL-1β + TNF-α + MaR1 + RvE1 group compared to IL-1β + TNF-α group: α-SMA increased 53%, tenomodulin increased 2.23-fold, periostin increased 76%).	(Albuquerque-Souza, E., 2020) [14]
Sprague Dawley rats, tooth extraction	MaR1, gelatin sponges as carriers to put into the sockets, and followed by topical use (twice a week)	0.5 μg/μL, 0.05 μg/μL	1. Accelerated wound closure (>33% increase), increased extraction socket bone fill (16% increase), preserved alveolar bone ridge (49% increase in both width and height) regulated M2-like macrophage surface marker CD206 (relative ratio decreased 0.26), and reduced post-operative pain scales.	(Wang, C.W., 2020) [15]
**1.2 Cardiovascular system**				
**Preclinical models**	**Administration of maresins**	**(Optimal) concentration**	**Efficacy**	**References**
Endotoxemia (LPS), C57BL/6 mice	MCTR1, *i.p.*	0.15 or 0.3 nmol/mouse (no significant difference)	1. Improved mitochondrial biogenesis and function (MCTR1 restored the expression of Sirt-1 total and nuclear protein, ~80% increased and ~40%, respectively).	(Yang, Y., 2020) [16]
	MCTR1, *i.v.*	0.15 nmol/mouse	1. Alleviates neutrophil infiltration (percentage of CD11b^+^Ly6G^+^ neutrophil population in the hearts reduced ~50%).	(Yang, Y., 2021) [17]
C57BL/6 mice, under the stimulation of LPS	MaR1(pretreatment), *i.p.*, followed by booster injections	100 ng and booster injections: 100 ng every 2 days/mouse	1. Protects the heart from injury and dysfunction (expression of cardiac injury marker lactate dehydrogenase (LDH) decreased ~20%, kinase isoenzyme (CK-MB) decreased ~53%).	(Li, D., 2021) [18]
Sepsis model (cecal ligation and puncture (CLP)), BALB/c mice	MaR1, *i.v.*	1.1 ng	1. Protecting mice receiving cecal ligation and puncture (CLP) from lung injury (histopathological scores reduced ~37.5%). 2. Improving liver and kidney function (level of alanine transaminase (ALT), aspartate transaminase (AST), creatinine (Cre), and blood urea nitrogen (BUN) in serum decreased ~20–50%). 3. Inhibiting production of inflammatory cytokines (IL-6, TNF-α, IL-1β decreased ~30–50%), growth of bacteria colonies, and activation of nuclear factor kappa B (NF-κb) pathway.	(Li, R., 2016) [19]
CLP, male C57B6/L mice	MaR1, *i.p.*	10 nM	1. Reduced the release of inflammatory cytokines in the plasma of sepsis mice (TNF-α, IL-1β, MPO, MIP-2, and IL-10 decreased ~20–30%). 2. Enhanced bacterial clearance and modulated immune cells (CFU decreased ~40%, macrophages increased ~40%, neutrophils decreased ~40%). 3. Attenuated mitochondrial dysfunction by regulating ROS associated with ALX and cAMP (ALX antagonist BOC-2 reversed the effects of MaR1).	(Gu, J., 2018) [20]
SuHx (Sugen 5416 injection and hypoxia exposure)-induced PAH model, male C57BL/6 mice	MaR1, *i.p.*, followed by booster injections	1 μg/mouse and booster injections: 100 ng every 2 days/mouse	1. Lessening right ventricular systolic pressure (RVSP), attenuating right ventricular dysfunction (RVD), reversing abnormal changes in pulmonary vascular remodeling, and inhibiting abnormal pulmonary artery smooth muscle cells’ (PASMCs’) proliferation (enhanced apoptosis of α-SMA positive cells, decreased phosphorylation of STAT3, AKT, ERK, and FoxO1 via LGR6).	(Li, H., 2022) [21]
Fed with high-fat diet (HFD), Apoe^−/−^ C57Bl/6 mice.	MaR1 and RvD2, *i.p.*, (additional 4 weeks of HFD feeding)	MaR1: 100 ng, RvD2: 100 ng, every second day	1. Preventing atheroprogression (decreased in necrotic core size ~30%, mac2 positive cells/plaque ~35%, vulnerability plaque index decreased ~47%; increased in total collagen ~33%, fibrous cap thickness ~20%, SMCα positive cells/plaque ~55%). 2. MaR1 and RvD2 induced a pro-resolving macrophage phenotype (increased CD206 positive macrophage ~4-fold, fold change in aortic RNA *Retnla* increased ~70% and *Nos2* decreased ~40%, decrease in TNF-α ~40% and IL-6 ~50%, increase in TGF -β~60%).	(Viola, J.R., 2016) [22]
Topical elastase induced abdominal aortic aneurysm (AAA) model, C57BL/6 wild-type (WT) mice, and TGF-β2 receptor knockout (SMC-TGFβr2−/−) mice	MaR1, *i.p.*	4 ng/g or 40 ng/g bodyweight	1. Reducing abdominal aortic aneurysm (AAA) growth of smooth muscle cells mediated by LGR6 receptors (decreased ~28% in aortic diameter, expression of TGF-β2 increased ~3.1-fold, MMP2 decreased ~48%, smooth muscle alpha actin (SM-αA) increased ~34%, efferocytosis of SMC increased ~2.69-fold at 7 d/~1.53-fold at 14 d).	(Elder, C.T., 2021) [23]
Myocardial infarction (MI) model by left anterior descending (LAD) coronary artery ligation, male C57BL/6 mice	MaR1, *i.p.*	10 ng/g every 2 days for 28 days	1. Improving cardiac function (increased LVEF ~1.73 fold and FS ~4.8 fold, and decreased left ventricular end-diastolic volume (LVEDV) ~59%, left ventricular end-systolic volume (LVESV) ~71%, left ventricular inner dimension at end-diastolic stage (LVIDd) ~29%, and left ventricular inner dimension at end-systolic stage (LVIDs) ~37%), attenuating ventricular structural remodeling (remaining 52% of myocardial fibrosis area), decreasing ventricular electrical remodeling (decreased action potential duration (APD)90 ~37%, APD50 ~37%, electrical alternans (ALT) threshold ~25%, suppressed the decrease in effective refractory period (ERP)) and myocardial apoptosis. 2. Alleviating cardiac oxidative stress after MI by activation of NRF2/HO-1 signaling (reduced the expression of fibrotic markers and malondialdehyde (MDA) level ~17%, increased Cx43 expression ~2.5 fold and serum superoxide dismutase (SOD) level ~1.6 fold) and inhibition of TLR4/NF-kB signaling (decreased TLR4, p-p65, TNFα, and IL-6 protein levels ~30–50%, and macrophage infiltration ~36%).	(Wang, F., 2022) [24]
**1.3 Digestive system**				
**Preclinical models**	**Administration of maresins**	**(Optimal) concentration**	**Efficacy**	**References**
Diet-induced obese (DIO) mice	MaR1, oral gavage	50 μg/kg	1. Relative abundance of *P. xylanivorans* increased. 2. IL-1β and TNF-α decreased.	(León, I.C., 2020) [25]
Dextran sulfate sodium (DSS) and 2,4,6-trinitrobenzene sulfonic acid-induced colitis mice	MaR1, e.v.	acute protocol: 0.1, 0.3, and 1 μg/animal chronic protocol: 0.3 μg/animal	1. The disease activity index improved. 2. Body weight and colonic tissue damage reduced.	(Marcon, R., 2013) [26]
BALB/c mice	MaR1, *i.p.*	0.03, 0.3, and 1 μg/animal	1. MaR1 showed antioxidative and anti-inflammatory effects, attenuating hepatic injury, oxidative stress, and lipid peroxidation.	(Li, R., 2016) [27]
Diethylnitrosamine (DEN)-induced liver fibrosis rat	MaR1, *i.p.*	4 ng/g	1. The aspartate transaminase (AST) concentration decreased by about 46%. 2. The alanine transaminase (ALT) concentration decreased by about 44%. 3. The hepatic index decreased by about 12%. 1. The pro-inflammatory cytokines TNF-α and IL-1β were increased by 6- and 5.5-fold, respectively, in relation to the control group, and 4.8- and 2.4-fold, respectively, in relation to the MaR1 + DEN group. 2. The anti-inflammatory IL-10 of the MaR1 + DEN group was 3.5- and 3.4-fold, respectively, compared to the control and MaR1 group, and 5.5-fold compared to the DEN group.	(Rodríguez, M.J., 2021) [28]
Diet-induced obese (DIO) mice	MaR1, *i.p.*/oral gavage	2 μg/kg (*i.p.*) or 50 μg/kg (oral gavage)	1. MaR1 decreased lipogenic enzymes and liver triglycerides content.	(Laiglesia, L.M., 2018) [29]
High-fat diet-induced hepatic steatosis mice	MaR1, *i.p.*	35 μg/kg	1. MaR1 ameliorated obesity-related liver steatosis by suppressing ER stress.	(Jung, T.W., 2018) [30]
High-fat diet-induced non-alcoholic steatohepatitis (NASH)	MaR1, *i.p.*	5 μg/kg	1. MaR1 enhanced the retinoic acid-related orphan receptor α (RORα) ability to activate the M2 polarity of liver macrophages, protecting the liver from NASH.	(Han, Y.H., 2019) [31]
Liver ischemia-reperfusion injury mice	MaR1, *i.p.*	4 ng/g	1. MAI (mitotic index) activity of hepatocytes was characterized by an intense cell division with 3.7- and 5.25-fold increases in the MaR1-sham and MaR1-IR groups, respectively. MaR1-IR showed an increase of 41% in cell division related to MaR1-sham livers. 2. IL-6 was increased 1.4 times in the MaR1-IR group compared to IR groups. Serum IL-6 was elevated 2.1 times in MaR1-sham with respect to the control and was 0.2 and 6 times less than the IR and MaR1-IR groups, respectively. 3. The increase in nuclear Nrf2 of the MaR1-IR group was more than 7-fold compared to the control.	(Soto, G., 2020) [32]
Lipopolysaccharide/d-galactosamine (LPS/D-GalN)-induced acute liver injury mice	MaR1, *i.p.*	50, 100 ng	1. MaR1 attenuated acute liver injury by ameliorating inflammation.	(Yang, W., 2022) [33]
Caerulein-induced pancreatitis mice	MaR1, *i.p.*	0.1, 0.5, 1 μg	1. MaR1 decreased serum levels of amylase, lipase, and inflammatory cytokines such as TNF-α, IL-1β, and IL-6.	(Lv, C., 2019) [34]
Cerulean-induced pancreatitis	MaR1, *i.p.*	1.0 ng	1. MaR1 alleviated inflammation of the pancreas and lungs by inhibiting the activity of NF-κB.	(Munir, F., 2019) [35]
**1.4 Endocrine system**				
**Preclinical models**	**Administration of maresins**	**(Optimal) concentration**	**Efficacy**	**References**
High-fat diet-induced obese C57BL/6J mice	MaR1, *i.p.*	2 μg/kg, 10 days	1. Reduced subcutaneous depot weight by ~18%, serum white adipose tissue (WAT)-secreted lectin by ~21%, and fasting glucose by ~13%.	(Martínez-Fernández, L., 2017) [36]
Leptin-deficient *ob*/*ob* mice		2 μg/kg, 20 days	1. Reduced ~15% basal glucose in insulin tolerance tests (ITT). 2. Increased *Glut-4* expression. 3. Reduced *Dpp-4* expression.	
High-fat diet-induced obese C57BL/6J mice	MaR1, oral gavage	50 μg/kg, 10 days	1. Reversed ~50% diet-induced increase in fasting glycemia. 2. Reduced ITT glucose levels by ~33%. 3. Partially restored muscle insulin-induced Akt phosphorylation.	(Martinez-Fernandez, L., 2021) [37]
Lean C57BL/6J mice	MaR1, *i.p.*	50 μg/kg, 3 h	1. Improved Akt phosphorylation in skeletal muscle and epididymal WAT.	
High-fat diet-induced obese C57BL/6J mice	MaR1, oral gavage	50 µg/kg, 10 days	1. Reversed high-fat diet-induced modulation of FGF-21 expression.	(Martinez-Fernandez, L., 2019) [38]
High-fat diet-induced obese C57BL/6J mice	MaR2, *i.p.*	5 μg/kg, 28 days; 10 μg/kg, 26 days	1. Downregulated plasma TNF-α levels and liver pro-inflammatory gene expression. 2. Liver weight, triglyceride levels, lipogenic gene expression, steatosis, and ALT, were not altered in 10 μg/kg (26 days) treatment.	(Sugimoto, S., 2022) [39]
**1.5 Immune system**				
**Preclinical models**	**Administration of maresins**	**(Optimal) concentration**	**Efficacy**	**References**
*Escherichia coli (E. coli)*-induced peritonitis, FVB mice	A panel of MCTR3, PCTR3, RCTR3 (non-target siRNA-injected), *i.p.*	50 ng each	1. Reduced PMN numbers in the exudate by ~70% and TNF-α protein level by ~50%.	(Chiang, N., 2021) [40]
Regenerative model, planaria	MCTR3, suspended in water	10 nM	1. Planaria regeneration index increased by ~50% (CTR/ctrl).	
Peripheral blood mononuclear cell (PBMC)-derived human macrophages	MCTR3, suspended in PBS^+/+^	10 nM	1. Enhanced phagocytosis of *E. coli* by ~50% at 60 min.	
PBMC-derived human macrophages	MaR1, suspended in PBS^+/+^	0.01 nM	1. Resulted in ~90% *E. coli* phagocytosis.	(Colas, R.A., 2016) [41]
		22-OH-MaR1(1 pM) and 14-oxo-MaR1(1 pM)	1. Resulted in ~75% and ~25% *E. coli* phagocytosis, respectively.	
Primary human monocytes, under the stimulation of LPS and engagement of TLR4	MaR1, suspended in RPMI	1.0–3.0 μM	1. Reduced ~50% release of TNF, IL-8, IL-1β, IL-12 p40.	(Gu, Z., 2016) [42]
		1 μM	1. Doubled IL-10 expression.	
PBMC-derived macrophages, under *Mycobacterium tuberculosis* infection	MaR1, suspended in RPMI	150 nM	1. Lowered intracellular bacterial burden by ~36% and TNF-α by more than 80%. 2. Increased bactericidal/permeability-increasing protein (BPI) expression by 66.5%.	(Ruiz, A., 2019) [43]
Primary human peripheral blood mononuclear cells, under stimulation of LPS	MaR1, suspended in DMEM	optimal: 100 nM	1. Reduced ~50% of TNF-α, IL-6, IL-1β mRNA and protein levels.	(Wang, W., 2021) [44]
Human PBMC-purified CD8^+^ and CD4^+^ T cells	MaR1, suspended in X-VIVO 15 mediums	10 nM	1. Downregulated cytokines.	(Chiurchiu, V., 2016) [45]
Human PBMC-purified anti-CD3/CD28-stimulated T cells			1. Reduced IL-2 production ~50%.	
Human PBMC-purified naïve CD4^+^ cells			1. Reduced differentiation into Th1 or Th17 cells and favored differentiation into Treg cells.	
**1.6 Integumentary/exocrine system**				
**Preclinical models**	**Administration of maresins**	**(Optimal) concentration**	**Efficacy**	**References**
UVB-induced skin inflammation model, hairless (HRS/J) or LysM-eGFP C57BL/6 background mice	MaR1, *i.p.*	10 ng/mouse, 10 min before UVB irradiation	1. Reduced skin edema manifested by a decrease in skin weight by ~37%. 2. Decreased neutrophil recruitment, keratinocyte apoptosis, epidermal thickness, MMP-9 activity, and collagen degradation.	(Cezar, T.L.C., 2019) [46]
Psoriasis model (imiquimod or IL-23 administration), C57BL/6 mice	MaR1, topical	100 ng in 20 μg ethanol/ear	1. Ameliorated ear swelling by ~40–50%. 2. Reduced epithelial thickness by ~33–50%. 3. Decreased dermal edema and a number of CD45^+^ cells and Ly-6G^+^ cells.	(Saito-Sasaki, N., 2018) [47]
Primary human adipocytes, under the stimulation of TNF-α	MaR1	10 nM	1. Reversed TNF-α-induced chemerin gene expression and protein secretion back to basal level.	(Sáinz, N., 2020) [48]
**1.7 Musculoskeletal system**				
**Preclinical models**	**Administration of maresins**	**(Optimal) concentration**	**Efficacy**	**References**
Aged (24-month-old) mice underwent tibial fracture	MaR1, *i.p.*	5 µg/kg	1. MaR1 decreased the percentage of pro-inflammatory macrophages (~52%) and serum levels of inflammatory cytokines IL-6 (~64%), IL-10 (~52%), TNFα (~60%). 2. MaR1 treatment also increased the bone volume (BV) within the fracture callus (~38%) and the relative amount of bone within the fracture callus. The ratio of bone volume and total volume increased by about 40%. 3. Bone content was higher in MaR1-treated samples. It increased by about 28%.	(Huang, R., 2020) [49]
Collagen-induced arthritis mice	MaR1, *i.p.*	0, 20, and 100 ng	1. Intervention of MaR1 improved the imbalanced Treg/Th17 ratio. MaR1 increased Treg cell proportion while reducing Th17 cell proportion dose dependently.	(Jin, S., 2018) [50]
**1.8 Nervous system**				
**Preclinical models**	**Administration of maresins**	**(Optimal) concentration**	**Efficacy**	**References**
Alzheimer’s disease model (bilateral hippocampal Aβ injection), C57BL/6 mice	MaR1, *i.c.v.*	0.01 µg	1. Improved mice performance in Morris Water Maze (MWM) by reducing escape latency by ~50% and increased numbers of platform crossing and time spent in the target quadrant by ~2-fold.	(Yin, P., 2019) [51]
Alzheimer’s disease model, *App^NL-G-F/NL-G-F^* mice	SPM-combined solution (RvE1, RvD1, RvD2, MaR1, and NPD1), intranasal delivery	40 ng per LM, three times a week for 9 weeks	1. Reduced microgliosis and recovered 57% of gamma oscillation power.	(Emre, C., 2022) [52]
Perioperative neurocognitive disorder model (orthopedic surgery), C57BL/6 and Ccr2^RFP/+^Cx3cr1^GFP/+^ mice	MaR1, *i.p.*	100 ng	1. Reversed surgery-reduced freezing time by ~20% in contextual fear conditioning.	(Yang, T., 2019) [53]
Neonatal Sprague Dawley rats, under exposure to sevoflurane	MaR1, *i.p.*	10 nM, 3 days	1. Improved MWM performances by reducing escape latency by ~75% and increasing platform crossing times, swimming distances, and staying time in the target zone, all by ~60%.	(Wu, Y., 2022) [54]
Chronic cerebral hypoperfusion model (2-vessel occlusion), Sprague-Dawley rats	MaR1, *i.t.*	0.05 μg	1. Decreased escape latency in MWM by ~50% maximally. 2. Alleviated blood–brain barrier (BBB) damage.	(Li, T., 2022) [55]
Brain ischemia/reperfusion injury model (middle cerebral artery occlusion), C57BL/6 mice	MaR1, intracerebroventricular (i.c.v.) injection	1 ng	1. Decreased ~40% of original infarct volume and reduced ~4% of brain water content in ischemic ipsilateral hemispheres. 2. Lowered neurological severity scores by ~50% and ~66% at 48 h and 72 h, respectively, after perfusion.	(Xian, W., 2016) [56]
Experimental autoimmune encephalomyelitis model, C57BL/6 mice	MaR1, *i.p.*	1 μg, 21 days	1. Lowered average EAE scores by ~40%. 2. Prevented ~72% area of myelin loss.	(Sánchez-Fernández, A., 2022) [57]
Spinal cord injury model, C57BL/6 mice	MaR1, *i.v.*	1 μg, 7 days	1. Raised Basso Mouse Scale scores by ~30%. 2. Reduced gait symmetry scores by ~26% and stance/width stepping variability scores by ~50%. 3. Elevated myelinated axons by ~20%.	(Francos-Quijorna, I., 2017) [58]
Spinal muscular atrophy model, colony bred from a pair of heterozygous SMNΔ7 mice	MaR1, *i.p.*	1 mg/kg, 11–13 days	1. Decreased righting reflex latency by ~66% and negative geotaxis test latency by ~50%.	(Ohuchi, K., 2018) [59]
Acute (carrageenan-induced) and chronic (complete Freund’s adjuvant (CFA)-induced) inflammatory pain model, Swiss, and LysM-eGFP mice	MaR1, *i.t.*	optimal: 10 ng	1. Alleviated acute and chronic inflammatory pain in mice by reducing the difference in withdrawal thresholds between baseline (at zero-time) and after 1–5 h carrageenan stimulation under stimulation of mechanical allodynia (by max:~2 g) and thermal hyperalgesia (by max:~5 s)	(Fattori, V., 2019) [60]
CFA-induced overt pain model, Swiss, and LysM-eGFP mice	MaR1, *i.t.*	10 ng	1. Reduced flinches and time spent licking the paw by ~50%.	
LPS-induced mechanical and thermal hyperalgesia models, Swiss mice	MaR2, *i.p.*	optimal: 30 ng	1. Lowered withdrawal by ~66% maximally in von Frey tests. 2. Increased latency by ~20–50% in hot plate tests. 3. Increased injured/non-injured paw weight ratio by ~25%.	(Fattori, V., 2022) [61]
Neuropathic pain model (spinal nerve ligation), Sprague Dawley rats	MaR1, *i.t.*	100 ng/10µL	1. Raised ipsilateral mechanical withdrawal threshold by ~50% and thermal paw withdrawal latency by ~50%.	(Gao, J., 2018) [62]
Radicular pain model (non-compressive lumbar disc herniation), Sprague Dawley rats	MaR1, *i.t.*	optimal: 100 ng	1. Attenuated neuropathic pain 2. Reversed ~50% of mechanical stimulus-induced reduction in paw withdrawal threshold. 3. Reversed ~75% of thermal stimulus-induced paw withdrawal latency.	(Wang, Y.H., 2020) [63,64]
Peripheral nerve injury model (sciatic nerve crush), ICR mice	MaR1 was applied onto damaged nerves using a hemostatic gelatin sponge	500 ng	1. Reduced gastrocnemius atrophy by preventing ~20% loss of gastrocnemius muscle weight ratio (ipsilateral/contralateral). 2. Promoted functional recovery more effectively than nerve growth factor in rotarod, von Frey, and Hargreaves tests.	(Wei, J., 2022) [65]
	MaR1, *i.t.*	optimal: 100 ng	1. Mitigated neuropathic pain. 2. Reversed ~66% of mechanical allodynia-induced reduction in paw withdrawal threshold. 3. Reversed ~80% of thermal hyperalgesia-induced paw withdrawal latency.	
	MaR1, intraplantar injection	50 ng	1. As compared to the nerve growth factor, MaR1 did not lower the pain thresholds.	
Fracture-associated post-operative pain model (tibial fracture and surgery), CD1 mice	MaR1 as peri- (500 ng, *i.v.*) and post-operative treatments (500 ng, *i.t.*)		1. Inhibited fracture-associated post-operative pain. 2. Reversed ~55% of mechanical allodynia-induced reduction in paw withdrawal threshold. 3. Reversed ~60% of mechanical allodynia-induced increase in paw withdrawal frequency. 4. Reversed ~82% of cold allodynia-induced increase in cold score.	(Zhang, L., 2018) [66]
Persistent allodynia dissociated from clinical arthritis signs (K/BxN serum-transfer model), C57BL/6 mice	MaR1, *i.p.*	100 ng, repeated injections	1. Induced amelioration of pain with later onset and longer duration. 2. Reversed ~66% of mechanical hypersensitivity-induced reduction in paw withdrawal threshold.	(Allen, B.L., 2020) [67]
**1.9 Reproductive system**				
**Preclinical models**	**Administration of aresins**	**(Optimal) concentration**	**Efficacy**	**References**
Peritonitis mice	MaR1, *i.p.*	50 ng	1. Human milk shortened the resolution interval in mouse peritonitis, and the magnitude of PMN infiltration of MaR1 is 76% and 58%.	(Arnardottir, H., 2016) [68]
Vulvar pain mice	MaR1, *i.p.*	1 μg/day, 4 weeks	1. MaR1 decreased sensitivity by increasing the pain threshold and suppressed PGE2 levels.	(Falsetta, M.L., 2021) [69]
**1.10 Respiratory system**				
**Preclinical models**	**Administration of maresins**	**(Optimal) concentration**	**Efficacy**	**References**
Human and mice precision-cut lung slices	MCTRs, *i.v.*	10 ng	1. MCTRs promote the resolution of allergic lung inflammation. 2. MCTRs blocked airway contraction in human precision-cut lung slices.	(Levy, B.D., 2020) [70]
BALB/c mice	MaR1, *i.p.*	0.1, 1, 10 ng	1. MaR1 suppressed the activation of the NF-κB signaling pathway, thus reducing COX-2 and ICAM-1, preventing inflammatory cell infiltration in the bronchoalveolar lavage fluid and excessive mucus production.	(Ou, G., 2021) [71]
Lipopolysaccharide (LPS)-induced acute lung injury mice	MCTR3	2 ng/g	1. Maresin inhibited cell death, inflammatory cytokine levels, and oxidative stress through the inactivation of the ALX/PINK1-mediated mitophagy pathway, protecting against LPS-induced ALI.	(Zhuang, R., 2020) [72]
Lipopolysaccharide (LPS)-induced acute lung injury mice	MCTR1, *i.v.*	200 ng	1. Alveolar fluid clearance (AFC) rate increased by about 86% in the MCTR1 + LPS group compared to the LPS group. 2. MCTR1 significantly promoted AFC by upregulating epithelial sodium channel and Na^+^-K^+^-adenosine triphosphatase expression in vivo.	(Han, J., 2020) [73]
mice under the stimulation of inhalant dust exposure	MaR1, *i.p.*	0.1, 1 ng	1. MaR1 significantly decreased bronchoalveolar lavage neutrophil infiltration and intracellular adhesion molecule-1(ICAM-1) expression.	(Nordgren, T.M., 2015) [74]
Bleomycin-induced lung fibrosis mice	MCTR1, *i.p.*	1 μg, 100 ng, 10 ng	1. MCTR1 protected tissue from destroyed and enhanced survival rate best at the dose of 1 μg.	(Pan, J., 2021) [75]
**1.11 Urinary system**				
**Preclinical models**	**Administration of maresins**	**(Optimal) concentration**	**Efficacy**	**References**
Cyclophosphamide (CP)-induced bladder inflammation mice	MaR1, *i.p.*	25 μg/kg	1. MaR1 promoted epithelial wound/barrier repair and reduced bladder inflammation and bladder weight. 2. The percentage of scratch closure of the MaR1 group is twice that of the control group.	(Hughes, F.M., 2022) [76]
Diabetic kidney disease (DKD) induced by high-fat diet mice	MaR1, *i.p.*	4 μg/kg	1. MaR1 alleviated DKD and glucotoxicity-induced inflammation via LGR6-mediated cAMP-SOD2 antioxidant pathway.	(Li, X., 2022) [77]
Sepsis mice model prepared by the CLP (cecal ligation and puncture group) method	MaR1, *i.p.*	0.5, 1 ng	1. The 7-day survival rates of the control group, the cecal ligation and puncture (CLP) group, the MaR1 low dose (LD-MaR1, 0.5 ng) group, and the MaR1 high dose (HD-MaR1, 1 ng) group were 100%, 16.67%, 58.33%, and 75%, respectively. 2. Research showed that MaR1 significantly increased the 7-day survival rate of septic mice and the anti-inflammatory factor while reducing bacterial load and pro-inflammatory cytokines.	(Sun, S., 2019) [78]
Sepsis-associated acute kidney injury (SA-AKI) induced by CLP mice model	MCTR1, *i.v.*	200 ng	1. MCTR1 inhibited ferroptosis and elevated the expression of Nrf2. 2. The percent survival of the CLP group is about 50% of the MCTR + CLP group.	(Xiao, J., 2021) [79]
Mice model underwent ischemia of the left kidney for 45 min and nephrectomy of the right kidney	MaR1, *i.p.*	1.0 ng	1. The histologic score of the IRI + MaR1 group is about 66% that of the IRI group. 2. MaR1 remarkably mitigated renal IRI-induced inflammation and oxidative stress.	(Qiu, Y., 2019) [80]
